# Insights into copper sensing and tolerance in *Pneumocystis* species

**DOI:** 10.3389/fmicb.2024.1383737

**Published:** 2024-05-15

**Authors:** Aleksey Porollo, Steven G. Sayson, Alan Ashbaugh, Sandra Rebholz, Julio A. Landero Figueroa, Melanie T. Cushion

**Affiliations:** ^1^Center for Autoimmune Genomics and Etiology, Cincinnati Children’s Hospital Medical Center, Cincinnati, OH, United States; ^2^Division of Biomedical Informatics, Cincinnati Children’s Hospital Medical Center, Cincinnati, OH, United States; ^3^Department of Pediatrics, University of Cincinnati, Cincinnati, OH, United States; ^4^Department of Internal Medicine, University of Cincinnati, Cincinnati, OH, United States; ^5^Cincinnati Veterans Affairs Medical Center, Cincinnati, OH, United States; ^6^Department of Chemistry, University of Cincinnati, Cincinnati, OH, United States

**Keywords:** *Pneumocystis*, copper sensing, copper tolerance, metalloproteome, cuproproteins, host-pathogen interaction

## Abstract

**Introduction:**

*Pneumocystis* species are pathogenic fungi known to cause pneumonia in immunocompromised mammals. They are obligate to their host, replicate extracellularly in lung alveoli and thrive in the copper-enriched environment of mammalian lungs. In this study, we investigated the proteome of *Pneumocystis murina*, a model organism that infects mice, in the context of its copper sensing and tolerance.

**Methods and results:**

The query for copper-associated annotations in FungiDB followed by a manual curation identified only 21 genes in *P. murina*, significantly fewer compared to other clinically relevant fungal pathogens or phylogenetically similar free-living fungi. We then employed instrumental analyses, including Size-Exclusion Chromatography Inductively Coupled Plasma Mass Spectrometry (SEC-ICP-MS), Immobilized Metal Affinity Chromatography (IMAC), and Liquid Chromatography–Tandem Mass Spectrometry (LC–MS/MS), to isolate and identify copper-binding proteins from freshly extracted organisms, revealing 29 distinct cuproproteins. The RNA sequencing (RNA-seq) analysis of *P. murina* exposed to various CuSO_4_ concentrations at three temporal intervals (0.5, 2, and 5 h) indicated that significant gene expression changes occurred only under the highest CuSO_4_ concentration probed (100 μM) and the longest exposure duration (5 h). This stimulus led to the upregulation of 43 genes and downregulation of 27 genes compared to untreated controls. Quantitative PCR (qPCR) confirmed the expression of four out of eight selected upregulated genes, including three assumed transcription factors (PNEG_01236, PNEG_01675, and PNEG_01730) and a putative copper transporter (PNEG_02609). Notably, the three applied methodologies — homology-based annotation, SEC-ICP-MS/IMAC/LC–MS/MS, and RNA-seq — yielded largely distinct findings, with only four genes (PNEG_02587, PNEG_03319, PNEG_02584, and PNEG_02989) identified by both instrumental methods.

**Discussion:**

The insights contribute to the broader knowledge of *Pneumocystis* copper homeostasis and provide novel facets of host-pathogen interactions for extracellular pathogens. We suggest that future studies of *Pneumocystis* pathogenicity and copper stress survival should consider the entire spectrum of identified genes.

## Introduction

Copper is a biochemically versatile and indispensable trace element that plays a fundamental role in various biological processes (Festa and Thiele, 2011). This transition metal is a critical cofactor for a diverse array of enzymes, functioning as a catalytic center in redox reactions. Among its numerous roles, copper is essential for electron transfer processes in mitochondrial cytochrome c oxidase, which is vital for cellular respiration and adenosine triphosphate (ATP) production. Copper contributes to the organism’s antioxidant defense system by serving as a cofactor for Cu/Zn superoxide dismutase, an enzyme that neutralizes harmful superoxide radicals. The multifaceted involvement of copper in cellular activities is particularly pronounced in fungi, where it serves as an indispensable component for copper-dependent enzymes. Such enzymes are essential for virulence and antioxidant defense in pathogenic fungi (Gerwien et al., 2018). Copper homeostasis is intricately linked to the regulation of fungal growth, development, and pathogenicity (Smith et al., 2017).

Conversely, an excessive accumulation of copper beyond homeostatic requirements can result in toxicity in various scenarios. First, copper cytotoxicity stems from its redox-active nature and its capacity to oxidize lipids, nucleic acids, and proteins by generating reactive oxygen species (ROS) such as hydroxyl radicals through Fenton-like reaction (Valko et al., 2005). Second, copper has a higher affinity to proteins that normally bind zinc, iron, or other transition metals and can replace these trace metals at their protein binding sites (Waldron et al., 2009). Since numerous metalloproteins are involved in amino acid biosynthesis, DNA replication and repair, telomere maintenance, and other critical cellular functions, these proteins may be significant targets in copper toxicity due to the loss of catalytic function.

The dual nature of copper is harnessed by animals in their host-pathogen interactions to combat invasive threats. Lungs are the natural route of infection and the initial infectious niche in mammals for many infectious agents. Host innate immune cells, particularly macrophages in the lungs, actively accumulate and intricately compartmentalize copper as an effective defense against invaders. When encountering pathogens, macrophages engulf and confine these microorganisms within a specialized compartment known as the phagolysosome. This microenvironment is uniquely inhospitable, characterized by heightened levels of ROS, reactive nitrogen compounds, low pH, substantial copper concentrations, and proteases, all meticulously orchestrated to eradicate these intruders. High copper levels are also found in plasma, where copper is bound primarily to ceruloplasmin, but also available from albumin, extracellular Cu/Zn-superoxide dismutase (SOD), and plasma metallothioneins. In contrast, the host’s nutritional immunity strategies aim to deprive invading pathogens of copper (among other important trace metals). For instance, the invasion of the kidney by the fungal pathogen *Candida albicans* during the later stages of infection results in a reduction of total kidney copper. Similarly, infection by *Cryptococcus neoformans* in the brain is associated with clear signs of copper limitation stress in the fungus. Further details on the copper-related host defense tactics against invading pathogens can be found in the following reviews (Garcia-Santamarina and Thiele, 2015; Besold et al., 2016).

While most bacterial pathogens do not exhibit a need for cytoplasmic copper, fungal pathogens and humans share common Cu-binding proteins located in various cellular compartments, emphasizing a commonality in their Cu homeostasis mechanisms. For example, in response to elevated pulmonary Cu concentrations during infection, *C. neoformans* employs a time-dependent induction of the expression of two metallothionein (MT) genes, CMT1 and CMT2. These genes act as primary and redundant defense mechanisms, protecting *C. neoformans* from otherwise toxic Cu levels (Ding et al., 2011, 2013). In contrast to the MTs of *C. neoformans*, *C. albicans* relies on the Cu-exporting pump CRP1 to play a dominant role in Cu detoxification and Cu-related virulence in this fungal pathogen (Weissman et al., 2000). When faced with copper deprivation in the kidney, the yeast shifts to induce copper uptake through the fungal copper transporter CTR1 and transitions from expressing Cu/Zn Sod1 to Mn Sod3 (Li et al., 2015). Likewise, *C. neoformans* initiates the CUF1/CTR4 axis when access to copper is limited in the brain (Waterman et al., 2007). A similar strategy was found in another fungal pathogen, *Aspergillus fumigatus*, that responds to low copper through AfMac1 (a homolog to Mac1, a copper-responsive transcription factor in *Saccharomyces cerevisiae*) by upregulating the *ctrC* and *ctrA2* genes that encode high-affinity copper transporters (Cai et al., 2017). The fungus utilizes the copper efflux pump (CrpA) to detoxify excess copper (Cai et al., 2018). More details on copper homeostasis in *A. fumigatus* can be found in the review (Song et al., 2019). *Histoplasma capsulatum*, on the other hand, encounters dichotomous copper conditions during macrophage interaction, necessitating adaptive responses to fluctuating copper availability. Initially, at the stage of innate immunity, the fungus is exposed to an excess of copper, prompting upregulation of the copper efflux pump CRP1 (Moraes et al., 2024) and activation of antioxidant enzymes, including catalase B (CATB), peroxidase, and Cu/Zn SOD1 (Moraes et al., 2023), to mitigate ROS stress. As the infection progresses, at the stage of adaptive immunity, *H. capsulatum* transitions to coping with copper scarcity, implementing a strategy for high-affinity copper acquisition via the Ctr3 transporter. This uptake mechanism is modulated by the copper-sensitive transcriptional regulator Mac1 (Ray and Rappleye, 2022). For bacterial responses to copper, the reader is referred to the following reviews (Arguello et al., 2013; Becker and Skaar, 2014; Andrei et al., 2020).

The multifaceted role of copper, serving both as a host defense mechanism and a pathogen vulnerability, underscores the intricate and dynamic nature of host-pathogen interactions in the battle against infectious agents. While *Histoplasma* fungi or other microorganisms are engulfed by macrophages and cope with macrophage-imposed hostile environments such as phagosomes, *Pneumocystis* spp. are extracellular organisms that grow within the lung alveoli, and as such, we studied their coping mechanisms outside macrophages. Our study explores the adaptive strategies employed by the fungal *Pneumocystis* species to flourish in the copper-enriched environment of mammalian lungs. These host-obligate parasitic extracellular organisms, marked by significant gene loss relative to free-living fungi (Hauser et al., 2010; Porollo et al., 2014; Ma et al., 2016), notably lack homologs to the majority of known fungal copper-control proteins. Intriguingly, recent findings have demonstrated a remarkable increase in the viability of *P. jirovecii* organisms *ex vivo* with addition of copper(II) sulfate to the culture medium (Riebold et al., 2023). Consequently, it becomes imperative to unravel the mechanisms governing copper homeostasis in these pathogens, with the aims of refining culture conditions and identifying potential new drug targets. This study represents the first endeavor to shed light on how these pathogenic fungi sense and tolerate elevated copper levels.

## Materials and methods

### Sampling of pathogen organisms and host tissue

*P. murina* organisms for these studies were obtained from 6 to 8-week old Bagg Albino (BALB/c) mice (Charles River Wilmington, MA). The mice were handled in strict accordance with good animal practice, as defined by the Institutional Animal Care and Use Committee at the Veterans Affairs Medical Center, Cincinnati, OH. To safeguard against environmental exposure of *P. murina* and other microbes, mice were housed under barrier conditions with autoclaved food, acidified water, and bedding in sterilized shoebox cages equipped with sterile microfilter lids.

Mice were immunosuppressed by adding dexamethasone (4 mg/L) to acidified drinking water and infected with *P. murina* organisms isolated from previously infected mice and stored in liquid nitrogen. A dose of 2 × 10^6^ organisms / 50 μL was instilled by intranasal inoculation under anesthesia. The mice were continued on immunosuppression for 6 weeks and then euthanized by CO_2_ for isolation of organisms.

Lungs were removed, homogenized (gentleMACS™ Dissociator, Miltenyi Biotec, Auburn, CA), and filtered through a 40 μm pore mesh, and cells were recovered by centrifugation at 3400 rpm for 10 min. Host cells were removed by a 2^nd^ centrifugation at 250 rpm for 5 min. Slides were then prepared to enumerate organisms for the downstream CuSO_4_ treatment, as previously described (Cushion et al., 2010).

### Treatment with copper(II) sulfate

Two experiments were conducted to evaluate the impact of copper(II) sulfate on *P. murina*. Experiment 1, a short-term assessment, focused on differential gene expression in response to CuSO_4_ exposure. Experiment 2, a long-term study, aimed to determine the viability of *P. murina* under sustained CuSO_4_ exposure.

During the isolation process, *P. murina* were purified from lung cells by washing at least 3 times with phosphate buffered saline (PBS) as well as a short incubation period with 0.85% aqueous ammonium chloride, after which they were washed again with PBS. After rinsing with PBS, the cells were centrifuged to form a pellet, reconstituted in PBS for the next washing.

*P. murina* cultures were prepared at 2.5 × 10^6^ cells/mL using Roswell Park Memorial Institute (RPMI) 1,640 medium (Gibco, Grand Island, NY) supplemented with 10% fetal bovine serum (FBS) (Cytiva, Marlborough, MA), 1,000 U/mL penicillin, 1,000 μg/mL streptomycin (Gibco, Grand Island, NY), 1% minimum essential medium (MEM) vitamin solution, and 1% MEM non-essential amino acid solution (Gibco, Grand Island, NY). Cultures were distributed in quadruplicate into 48-well polystyrene plates (Corning Costar, Glendale, AZ). Each well was inoculated with 0.25 mL of either media (control) or CuSO_4_ solutions at final concentrations of 1, 10, and 100 μM for both experiments, and an additional concentration of 625 μM for Experiment 2. The plates were incubated at 36°C in a 5% CO_2_ atmosphere.

Sampling for ATP quantification was conducted at designated time points: 30 min, 2 h, and 5 h for Experiment 1; and 1, 3, 5, and 7 days for Experiment 2. For this, 50 μL samples were extracted from each well and transferred to a Nunclon Delta Surface white 96-well plate (Nunc A/S, Roskilde, Denmark). ATP levels were quantified using an ATP-lite luciferin-luciferase assay (Perkin Elmer, Waltham, MA), with luminescence readings obtained via a Synergy HTX spectrophotometer (BioTek, Winooski, Vermont). Background luminescence was measured in control wells with blank culture medium. To confirm sample purity, microscopic examinations for microbial contamination were performed and the cultures were microscopically monitored over the sampling periods to detect any bacterial or fungal outgrowth.

### Instrumental analysis

Cell pellets were lysed on ice using a lysis buffer composed of 0.1% SDS, 1% digitonin, 50 mM Tris–HCl, and 50 mM NaCl (pH 7.4) for 5 min, followed by 30 s of vortexing at maximum speed. The resulting cell lysates were then filtered through a 0.45 μm filter. Protein concentrations were determined using the Bradford assay, and the lysis buffer in each sample was adjusted accordingly to standardize protein levels across all samples. These lysates underwent SEC-ICP-MS analysis to detect copper across different molecular size fractions. The separation utilized a TSKgel G3000SWxl column (7.8 × 300 mm, 10 μm particle size, Tosoh Corporation, Tokyo, Japan) connected to an Agilent 1200 HPLC system. The mobile phase consisted of 50 mM ammonium acetate and 0.5% methanol (pH 7.4), with a flow rate of 0.6 mL/min. Copper quantification in each fraction employed three 50 μL injections, calibrated against a Cu standard curve (5 to 100 ng/mL) derived from human recombinant ceruloplasmin. Total metal content was assessed in triplicate aliquots via microwave digestion, comparing SEC-derived copper concentrations to total copper levels to ascertain a 94 ± 2% recovery efficiency.

Post-copper profiling, remaining samples (approximately 20 mL, excluding 100 μL for whole-lysate proteomics and 500 μL for non-fractionated IMAC enrichment) were fractionated via multiple 100 μL HPLC injections, with column performance verified every 20 injections using standards (Bio-Rad gel filtration standard mix and ceruloplasmin for Cu signal). Offline fraction collection from SEC was timed at intervals for high (10–12 min), medium (12.5–17.5 and 17.5–20 min), and low (20–25 min) molecular weight fractions. Collected fractions were lyophilized prior to IMAC processing.

To prepare for Cu-based IMAC protein enrichment, native copper was removed from proteins using Chelex 100 resin (Bio-Rad, CA) to expose Cu-binding sites. Proteins, either from whole lysate or dried fractions, were resuspended in 2 mL of 50 mM Na_2_HPO4 and 300 mM NaCl (pH 7.4), treated with 0.5 g of Chelex resin, and agitated at 4°C for 1 h, centrifuged at 5,000 g for 2 min. This process was repeated two more times to maximize copper removal. The clarified supernatant was then subjected to IMAC enrichment using the Cu-loaded IMAC resin.

An IMAC copper resin (Geno Technology, Inc., MO) was used to enrich the SEC-fractionated samples for Cu-binding proteins. The manufacturer instructions were followed, including the rinsing of the resin with milli-Q water, the conditioning of the resin with the loading buffer that contains 10 mM imidazole, loading of the sample followed by 20 min of agitation as incubation, the removal of the supernatant, washing of the resin with binding buffer, and the final step consisted on the elution of the Cu-binding proteins with 3 consecutive volumes of 50 mM Na_2_HPO4, 0.3 M NaCl, 0.25 M imidazole, pH 8.0. The amount of resin was adjusted according to the Cu concentration in each fraction, with the nominal Cu concentration of 30 μmol per ml of resin as reported by the vendor. As a positive control, Cu-depleted ceruloplasmin was introduced to the sample blank, while Zn-depleted carbonic anhydrase served as the negative control in a separate sample blank. The IMAC procedure was applied to the controls in a manner identical to that of the fractionated sample. The enriched sample fractions obtained were lyophilized to dryness and re-suspended in 50 mM ammonium carbonate for future tryptic digestion. Protein identification was conducted at the University of Cincinnati proteomics core facility utilizing liquid chromatography–tandem mass spectrometry (LC–MS/MS), following the previously published protocol (Green et al., 2013). Samples submitted for proteomics analysis included the high, medium, and low molecular weight fractions derived from the uninfected host lungs, the host lungs infected with *P. murina*, and the purified pathogen.

### RNA sequencing and analysis

*P. murina* organisms were resuspended in 500 μL of mirVana miRNA lysis buffer (Invitrogen, Waltham, MA) and promptly snap-frozen at −80°C for subsequent storage and transfer to a sequencing core. Directional polyA RNA sequencing (RNA-seq) was conducted at the Genomics, Epigenomics, and Sequencing Core at the University of Cincinnati, employing established protocols detailed in previous publications (Walsh et al., 2019; Rapp et al., 2020) with updates. Briefly, total RNA quality was assessed using the Bioanalyzer (Agilent, Santa Clara, CA). The NEBNext Poly(A) mRNA Magnetic Isolation Module (New England BioLabs, Ipswich, MA) was employed for the isolation of polyA RNA during library preparation, utilizing 400 ng of high-quality total RNA. The enrichment of polyA RNA was performed using the SMARTer Apollo automated NGS library prep system (Takara Bio United States, Mountain View, CA). Subsequently, the NEBNext Ultra II Directional RNA Library Prep kit (New England BioLabs) was utilized for library preparation, employing a Polymerase Chain Reaction (PCR) cycle number of 10. Following library QC and quantification via Qubit quantification (ThermoFisher, Waltham, MA), individually indexed libraries were proportionally pooled and subjected to sequencing on the NextSeq 550 Sequencer (Illumina, San Diego, CA) with a sequencing setting of SR 1 × 85 bp, generating approximately 25 million reads. Upon completion of the sequencing run, FASTQ files for subsequent data analysis were automatically generated through Illumina BaseSpace Sequence Hub.

Raw sequencing files were first preprocessed by FASTP (Chen et al., 2018) to remove or prune low quality reads, and trim adapter sequences. Then, transcript abundance was quantified by SALMON (Patro et al., 2017) using the *Pneumocystis murina* B123 strain’s genome as the reference (NCBI RefSeq Assembly ID: GCF_000349005). Differential gene expression analysis was conducted through DESeq2 (Love et al., 2014). Differentially expressed genes were identified based on a fold change (FC) of ≥2 and an adjusted *p*-value (*q*-value) of <0.05.

### RT-qPCR

Genes of interest were selected for validation by reverse transcription-quantitative polymerase chain reaction (RT-qPCR). The oligonucleotide primers to these genes were designed based on sequences from NCBI/GenBank. Primers were designed to span an intron junction to detect amplification of genomic DNA. cDNA was synthesized from the extracted RNA using SuperScript IV VILO master mix with ezDNase enzyme (Invitrogen, Grand Island, NY) per the manufacturer’s protocol. The remaining RNA and the freshly synthesized complementary DNA (cDNA) were stored at **−**80°C.

Real-time PCR was performed in an Applied Biosystems 7,500 fast real-time PCR system (Life Technologies, Grand Island, NY). The reactions were performed in triplicate in a final volume of 20 μL containing 1 μL PowerUp SYBR green master mix (Life Technologies, Grand Island, NY) and 500 nM each primer pair. The reaction mixtures were initially incubated at 50°C for 2 min and 95°C for 2 min, followed by 40 cycles of 15 s at 95°C, 59°C for 15 s, and 72°C for 1 min. Fluorescence data were captured during the 72°C step. Disassociation curves were also calculated for the reactions to determine the melting temperature (Tm) of the products. The reference gene used was thymidylate synthase (TS). The threshold cycle (ΔCT) value between the validation gene and the TS gene was calculated by subtracting the average CT value of TS from the average CT value of the validation gene. ΔΔCT values were calculated by subtracting the ΔCT value of the TS gene from the ΔCT value of the validation sample.

## Results

### Annotated copper related proteins in *Pneumocystis* species

Copper associated protein annotations were retrieved from the FungiDB database (Amos et al., 2022) for sequenced *Pneumocystis* species and other clinically significant and model fungi (as of August 2023). Since FungiDB transfers annotations inclusively, even from non-top homologs, an additional manual curation of *Pneumocystis* proteins was conducted using a homology search by BLAST against the NCBI nr database (top 250 hits with *E*-value<0.001, restricted to the fungi taxon) in conjunction with the UniProt and Pfam annotations. Table 1 provides a summary of the number of proteins identified using the keyword “copper.” Notably, *Pneumocystis* species exhibit approximately three to five times fewer copper-associated proteins compared to other fungi. Given that this study employed *P. murina* as the experimental model organism, Table 2 presents a list of respective proteins, derived from FungiDB annotations, either through direct gene description (second column) or annotations of homologous genes (third column), followed by the manual curation.

**Table 1:** Summary of copper-associated proteins in select clinically important and model fungi in FungiDB.

Organism	FungiDB
*Aspergillus fumigatus*	128
*Candida albicans*	115
*Cryptococcus neoformans*	76
*Histoplasma capsulatum*	84
*Saccharomyces cerevisiae*	109
*Schizosaccharomyces pombe*	57
*Pneumocystis canis*	12
*Pneumocystis carinii*	21
*Pneumocystis jirovecii*	16
*Pneumocystis murina*	21
*Pneumocystis oryctolagi*	15
*Pneumocystis wakefieldiae*	17

**Table 2:** Copper-associated proteins in *Pneumocystis murina*, as annotated in FungiDB.

Sequence ID^1^	Gene description	Annotation from orthologs^2^	*Pneumocystis* spp.^3^
PNEG_02820 M7NK89	Transcriptional activator HAP2	Ortholog(s) have role in cellular response to copper ion, cellular response to alkalinity (Hap2)	T552_01638 [*P. carinii*] MERGE_003017 [*P. wakefieldiae*]
PNEG_00941 M7NQ23	Copper-fist domain-containing protein	Copper-fist domain-containing protein	T552_00746 [*P. carinii*] T551_02441 [*P. jirovecii*] PORY_002473 [*P. oryctolagi*] MERGE_001952 [*P. wakefieldiae*]
PNEG_00378 M7PLM4	Transcription factor CBF/NF-Y/archaeal histone domain-containing protein	Ortholog(s) have role in cellular response to copper ion, cellular response to drug (Hap3)	PCANB_001520 [*P. canis*] T552_00479 [*P. carinii*] T551_01075 [*P. jirovecii*] PORY_000583 [*P. oryctolagi*] MERGE_000347 [*P. wakefieldiae*]
PNEG_00015 M7NWP0	Guanine nucleotide-binding protein subunit alpha	G-protein alpha subunit, regulates filamentous growth, copper resistance, involved in cAMP-mediated glucose signaling, reports differ on role in cAMP-PKA pathway, MAP kinase cascade, Gpr1 C terminus binds Gpa2, regulates HWP1 and ECE1 (Gpa1)	PCANB_001619 [*P. canis*] T552_01369 [*P. carinii*] T551_03530 [*P. jirovecii*] PORY_000017 [*P. oryctolagi*] MERGE_001611 [*P. wakefieldiae*]
PNEG_03449 M7P395	Cytochrome c oxidase assembly protein COX19	Copper chaperone (Cox19)	T552_03416 [*P. carinii*]
PNEG_03151 M7NIC4	Guanine nucleotide-binding protein alpha-2 subunit	G-protein alpha subunit, regulates filamentous growth, copper resistance, involved in cAMP-mediated glucose signaling, reports differ on role in cAMP-PKA pathway, MAP kinase cascade, Gpr1 C terminus binds Gpa2, regulates HWP1 and ECE1 (Gpa2)	PCANB_001619 [*P. canis*] T552_02231 [*P. carinii*] T551_00018 [*P. jirovecii*] PORY_000017 [*P. oryctolagi*] Pcg1 [*P. wakefieldiae*]
PNEG_00269 M7NX34	C2H2-type domain-containing protein	Ortholog(s) have role in cellular response to copper ion (Iec1)	PCANB_001939 [*P. canis*] T552_01119 [*P. carinii*] T551_00905 [*P. jirovecii*] PORY_001283 [*P. oryctolagi*] MERGE_002115 [*P. wakefieldiae*]
PNEG_00579 M7NV24	Copper transport protein	Copper transporter (Ctr1)	PCANB_000444 [*P. canis*] T552_02407 [*P. carinii*] T551_00219 [*P. jirovecii*] PORY_000332 [*P. oryctolagi*] MERGE_000968 [*P. wakefieldiae*]
PNEG_03174 M7PD09	Cytochrome c oxidase assembly factor 6	Ortholog(s) have copper ion binding activity, role in cellular copper ion homeostasis, mitochondrial cytochrome c oxidase assembly and mitochondrial intermembrane space localization (Coa6)	T552_02208 [*P. carinii*] T551_03213 [*P. jirovecii*]
PNEG_00316 M7NRE0	CBS domain-containing protein	Ortholog(s) have role in cellular copper ion homeostasis (Gef1)	T552_00540 [*P. carinii*] T551_03154 [*P. jirovecii*] PORY_002181 [*P. oryctolagi*] MERGE_000402 [*P. wakefieldiae*]
PNEG_02325 M7NQ40	Transmembrane 9 superfamily member	Ortholog(s) have role in cellular copper ion homeostasis, endosomal transport, invasive growth in response to glucose limitation, pseudohyphal growth, vacuolar transport (Tmn2)	PCANB_001305 [*P. canis*] T552_02880 [*P. carinii*] T551_01749 [*P, jirovecii*] PORY_000047 [*P. oryctolagi*] MERGE_000876 [*P. wakefieldiae*]
PNEG_03389 M7P2Z6	Copper transport protein 86	Copper transport protein 86 (Ctr86)	T552_03364 [*P. carinii*] PORY_001047 [*P. oryctolagi*] MERGE_001404 [*P. wakefieldiae*]
PNEG_03384 M7P2Y9	Copper-fist domain-containing protein	Copper fist DNA (Cup2 / Ace1)	PCANB_000685 [*P. canis*] T552_03369 [*P. carinii*] T551_01790 [*P. jirovecii*] PORY_001042 [*P. oryctolagi*] MERGE_001399 [*P. wakefieldiae*]
PNEG_03485 M7NHK0	Protein GZF3	Ortholog(s) have role in filamentous growth, cellular response to copper ion, cellular response to drug, cellular response to heat, cellular response to lithium ion (Gzf3)	PCANB_000644 [*P. canis*] T552_02999 [*P. carinii*] T551_02293 [*P. jirovecii*] PORY_002669 [*P. oryctolagi*] MERGE_001504 [*P. wakefieldiae*]
PNEG_00182 M7PMR1	COX assembly mitochondrial protein	Copper-binding protein of the mitochondrial inner membrane (Cmc1)	T552_01205 [*P. carinii*] PORY_000779 [*P. oryctolagi*] MERGE_002033 [*P. wakefieldiae*]
PNEG_00123 M7NWM3	Cytochrome c oxidase copper chaperone	Copper metallochaperone (Cox17)	PCANB_000100 [*P. canis*] T552_01264 [*P. carinii*]
PNEG_02634 M7NNL8	COX assembly mitochondrial protein	Copper-binding protein of the mitochondrial intermembrane space (Cmc1)	T552_00027 [*P. carinii*] T551_02800 [*P. jirovecii*] PORY_002626 [*P. oryctolagi*] MERGE_002857 [*P. wakefieldiae*]
PNEG_02013 M7NRE2	Cytochrome c oxidase assembly protein COX16, mitochondrial	Mitochondrial copper chaperone for cytochrome c oxidase (Cox16)	T552_01886 [*P. carinii*] T551_01903 [*P. jirovecii*] PORY_001903 [*P. oryctolagi*] MERGE_000496 [*P. wakefieldiae*]
PNEG_01400 M7NSM0	HMA domain-containing protein	Copper P-type ATPase (Pca1)	PCANB_000482 [*P. canis*] T552_01086 [*P. carinii*] T551_00173 [*P. jirovecii*] PORY_000376 [*P. oryctolagi*] MERGE_002331 [*P. wakefieldiae*]
PNEG_01457 M7NNB4	Thioredoxin domain-containing protein	Copper chaperone (Sco1)	PCANB_002293 [*P. canis*] T552_04099 [*P. carinii*] T551_02510 [*P. jirovecii*] MERGE_002284 [*P. wakefieldiae*]
PNEG_00544 M7PM36	Cytochrome c oxidase assembly protein CtaG/Cox11	Has domain(s) with predicted copper ion binding activity (Cox11)	PCANB_002185 [*P. canis*] T552_00312 [*P. carinii*] T551_03623 [*P. jirovecii*] PNEG_00544 [*P. murina*]

1 NCBI Gene database ID followed by the UniProt protein ID.

2 Copper-related annotation transferred from orthologs provided by FungiDB. Where possible, gene symbols of the orthologous proteins from model organisms are specified in parentheses.

3 NCBI Gene database IDs of the *P. murina* homologs in the other Pneumocystis species.

### Instrumentally identified copper-associated proteins in *Pneumocystis murina*

**Figure 1:**
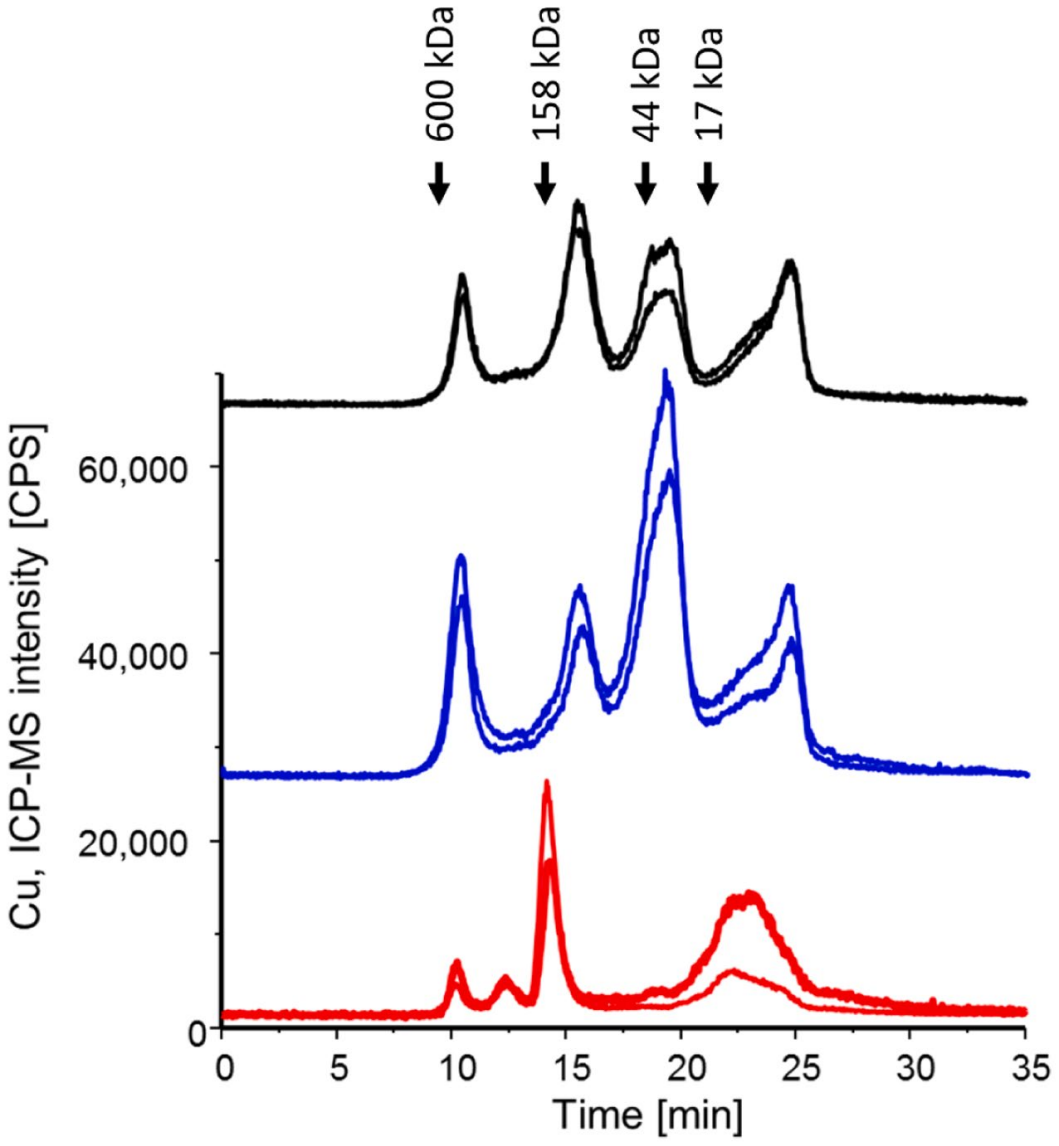
SEC-ICP-MS chromatograms were generated for *P. murina* samples (in red), lung homogenates from infected mice (in black), and lung homogenates from uninfected mice (in blue) to investigate the Cu metalloproteome. Each analysis was conducted in two technical replicates, and the chromatograms have been offset for clarity. The peaks observed at 23–24 min correspond to free copper. Molecular weight markers are indicated based on the Bio-Rad Gel filtration standard mixture containing thyroglobulin (bovine), g-globulin (bovine), ovalbumin (chicken) and myoglobin (horse).

*P. murina* cell lysates and lung homogenates from both infected and uninfected mice underwent SEC-ICP-MS analysis to detect Cu. Figure 1 presents the chromatograms for Cu profiling, where the area under the curve correlates directly with the Cu concentration in each fraction. Since the total protein concentration of each cell lysate was normalized, the chromatograms allow for a direct comparison of Cu content and distribution. Notably, the Cu distribution in host cell lysates remained consistent regardless of *P. murina* infection. However, the Cu distribution in the isolated pathogen markedly differed, underscoring the effectiveness of the pathogen isolation procedure.

A summary of the subsequent analysis of the fractions and the identification of the IMAC enriched Cu-binding proteins via LC–MS/MS is presented in Table 3. Of note, since the pathogen cannot be cultivated, any samples with extracted and purified organisms are still contaminated with the host cells. Table 4 provides the list of *P. murina* proteins identified in all molecular weight factions. The complete list of the identified proteins associated with copper, both for the pathogen and the host, can be found in Supplementary File S1.

**Table 3:** Counts of Cu-associated proteins in host and pathogen identified by proteomics.*

Sample source	Molecular weight fraction		
	Heavy (10–12 min)	Medium (12.5–17.5, 17.5–20 min)	Low (20–25 min)
Mouse non-infected	238/1	4/0	72/0
Mouse infected	196/13	3/0	140/9
*P. murina*	52/11	48/9	58/12

* First number is the count of host proteins, second – pathogen proteins.

**Table 4:** Cu-associated *P. murina* proteins identified by proteomics.

Sequence ID*	Gene description	Sequence ID	Gene description
PNEG_00053 M7NWD9	Ubiquitin-60S ribosomal protein L40	PNEG_02584 M7P5X2	Hsp60-like protein
PNEG_00081 M7NWG8	ATP synthase subunit alpha	PNEG_02587 M7NKU6	Hsp72-like protein
PNEG_00288 M7NVF6	Thioredoxin	PNEG_02593 M7PFP3	ADF-H domain-containing protein
PNEG_00446 M7NRP9	Uncharacterized protein	PNEG_02625 M7NJQ3	Malate dehydrogenase
PNEG_00557 M7PM51	Cytochrome c	PNEG_02855 M7NJ81	Peptidyl-prolyl cis-trans isomerase
PNEG_00617 M7NUN2	Eukaryotic translation initiation factor 5A	PNEG_02989 M7NNJ9	Msg2_C domain-containing protein
PNEG_00966 M7PK55	ATP synthase subunit beta	PNEG_03149 M7NMG4	Protein disulfide-isomerase
PNEG_01119 M7PIX8	ADP-ribosylation factor 1-like 2	PNEG_03319 M7NIR6	HATPase_c domain-containing protein
PNEG_01454 M7P8I8	COesterase domain-containing protein	PNEG_03333 M7NHW5	NTF2 domain-containing protein
PNEG_01591 M7P925	Protein disulfide-isomerase domain	PNEG_03379 M7P2Y0	CS domain-containing protein
PNEG_01876 M7PGV1	Glyceraldehyde-3-phosphate dehydrogenase	PNEG_03406 M7NI55	Chaperone DnaK
PNEG_02095 M7NQG2	Ubiquitin-like protein pmt3/smt3	PNEG_03434 M7PCV6	Uncharacterized protein
PNEG_02179 M7P6T2	Profilin	PNEG_03614 M7NM54	Uncharacterized protein
PNEG_02367; PNEG_04328 A0A0W4ZWW3	Aldo_ket_red domain-containing protein	PNEG_04005 A0A0W4ZX34	Msg2_C domain-containing protein
PNEG_02449 M7P5E7	Peptidyl-prolyl cis-trans isomerase		

* NCBI Gene database ID followed by the UniProt protein ID.

The proteomic identification through the IMAC enrichment method likely includes a range of metalloproteins typically linked with zinc, manganese, or other divalent metal ions, alongside the intended copper-binding proteins. This diversity arises from the demetallation process using Chelex 100 resin, which also extracts these metals while primarily removing Cu to generate apo-forms of the existing Cu-binding proteins. The predictability of differing affinities for Cu across various apo-forms of metalloproteins is challenging due to the IMAC resin’s nature and the attempt to limit available Cu to minimize nonspecific binding. Consequently, achieving complete selectivity for Cu-binding proteins is difficult. To enhance method specificity, optimization of incubation times, rinse volumes, and elution buffer volumes was undertaken to minimize the retention of carbonic anhydrase, a common off-target zinc protein, employing ceruloplasmin as a positive control due to its multifaceted copper-binding sites. Further refinement was evidenced by re-analyzing demetallated SEC fractions with ICP-MS prior to IMAC enrichment, revealing an 89 ± 6% reduction in the original Cu content (data not shown). This adjustment significantly improved the selectivity for ceruloplasmin over carbonic anhydrase, potentially decreasing false negatives in Cu-proteome analysis.

### Gene expression in *Pneumocystis murina* upon copper stimulus

To gain insights into the copper sensing mechanisms in *P. murina*, we exposed freshly extracted organisms to copper(II) sulfate for 0.5, 2, and 5 h, followed by RNA-seq analysis. In the initial batch, extracted from 6 mice, we applied concentrations of 1 ng/mL and 100 ng/mL of CuSO_4_ for comparison with untreated organisms. Notably, no statistically significant differential gene expression was observed (Supplementary Figure S1A).

In a subsequent batch, we exposed the organisms to elevated CuSO_4_ concentrations: 1, 10, and 100 μM (0.1596, 1.596, and 15.96 μg/mL, respectively), at the same time points as the first batch. In this case, organisms from three out of five animals exhibited differential gene expression patterns (Supplementary Figure S1B), leading to their selection for further RNA-seq analysis.

**Figure 2:**
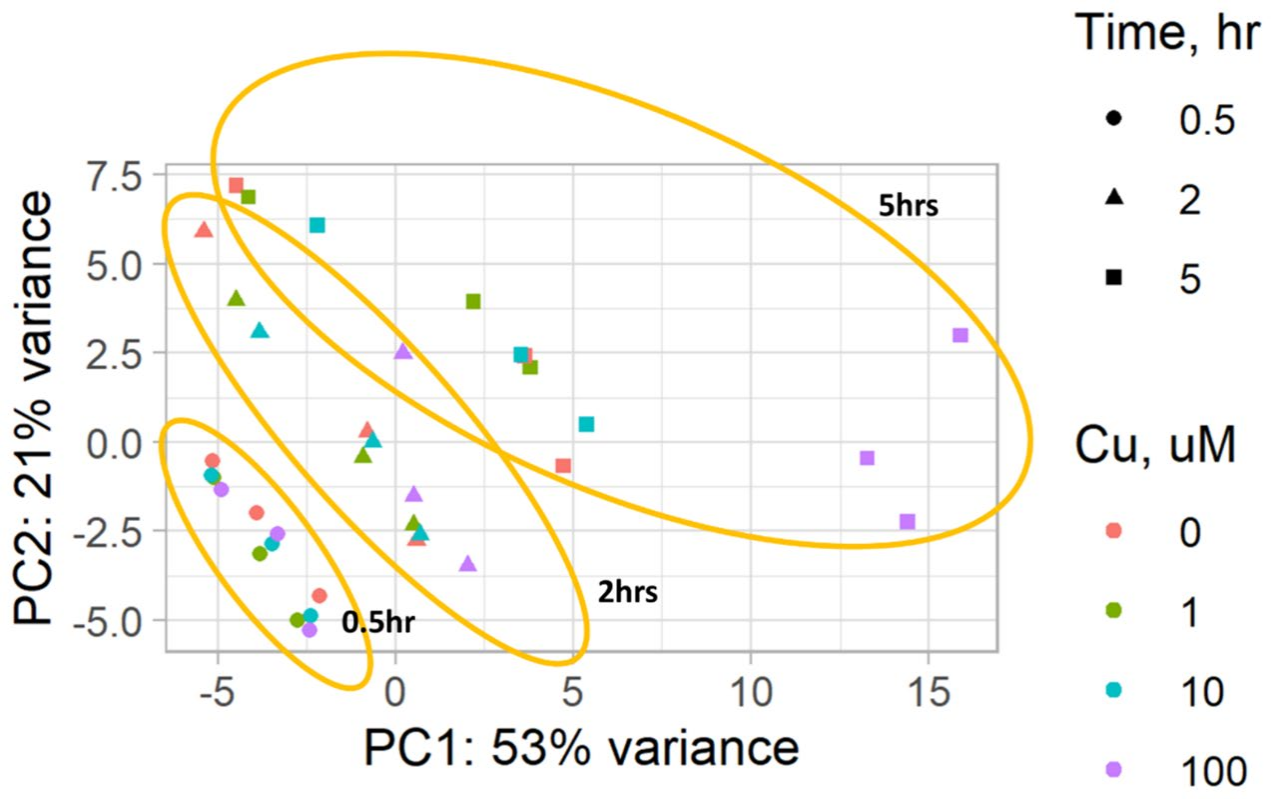
A PCA plot of the RNA-seq data from *P. murina* organisms exposed to CuSO_4_ at concentrations of 0 (controls), 1, 10, and 100 μM over time durations of 0.5, 2, and 5 h. Results are based on three biological replicates, i.e., each derived from organisms originating from three distinct animals. The PCA plot highlights the grouping of samples by the duration of exposure to CuSO_4_, marked in orange.

Figure 2 depicts the temporal dynamics of gene perturbations, with notable changes occurring at the 5-h time point. The most significant perturbation was evident during treatment with 100 μM CuSO_4_. Table 5 provides a summary of the genes that were statistically significantly perturbed. Comprehensive gene lists can be accessed in Supplementary File S2.

**Table 5:** Differentially expressed genes in *P. murina* upon CuSO_4_ treatment at the 5-h time point.

Comparison groups	Total perturbed genes	
	Upregulated	Downregulated
100 μM vs. Controls	43	27
100 μM vs. 1 μM	55	38
100 μM vs. 10 μM	40	28

**Figure 3:**
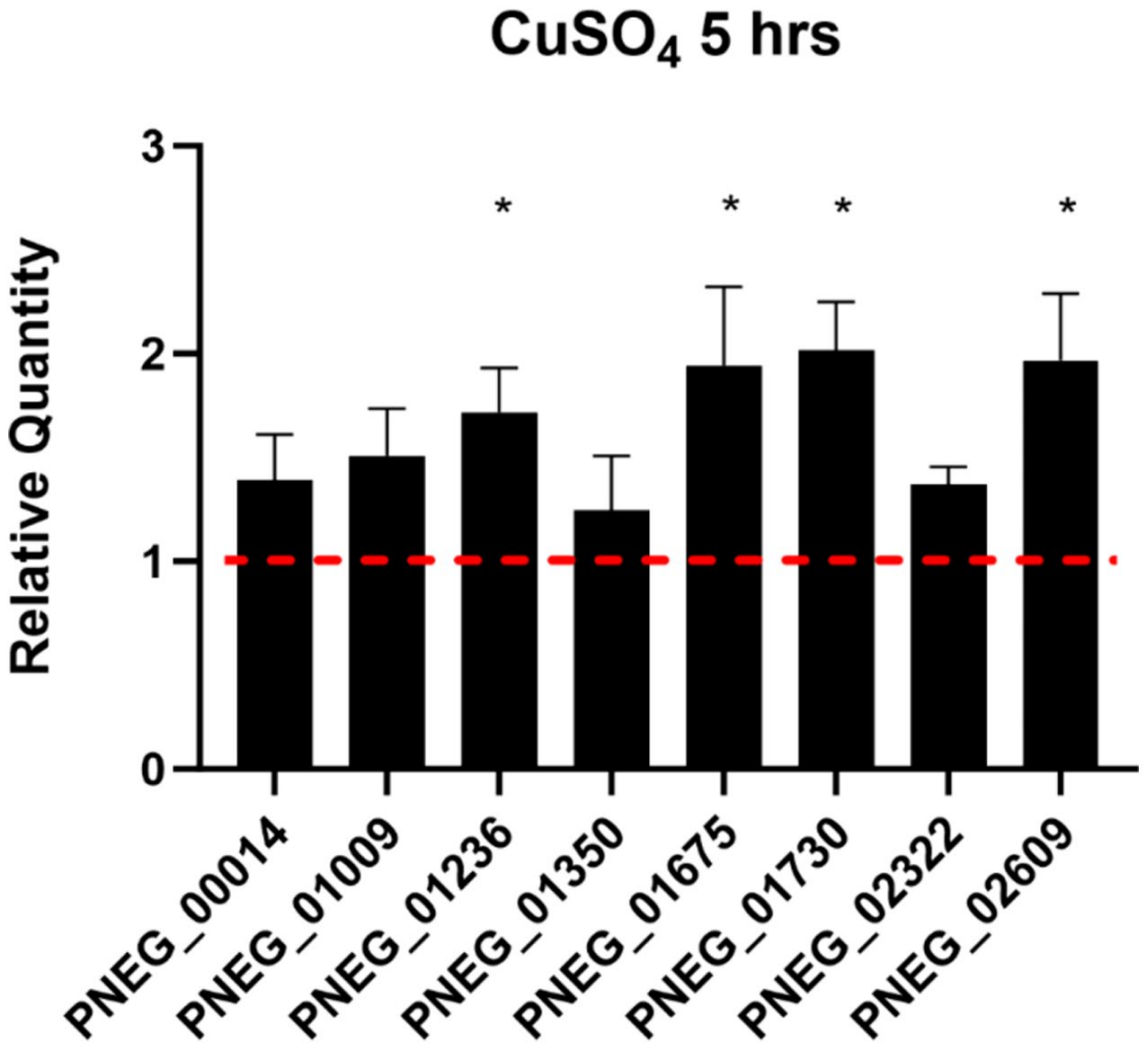
RT-qPCR of select genes found over-expressed in *P. murina* treated with 100 μM CuSO4 at 5 h. Reference gene used is thymidylate synthase. Measurements were made in triplicates. Relative quantity is shown as 2ΔΔCT. * – statistical significance at *p*-value <0.05.

To validate some genes as potential candidates responding to copper excess in *P. murina*, we selected eight genes with descriptions or domain annotations related to copper or other metal ions (Table 6). Figure 3 illustrates the outcomes of RT-qPCR analysis for these genes using samples treated with 100 μM CuSO4, in comparison to control samples. Notably, Figure 3 reveals that four out of the eight selected genes exhibit statistically significant upregulation. Among these four genes, PNEG_02609 is indicative of a metal ion transporter, while PNEG_01236, PNEG_01675, and PNEG_01730 are likely transcription factors.

**Table 6:** *P. murina* genes selected for RT-qPCR validation.

Gene ID Primer sequences	Gene description and family/domain annotation
PNEG_02609 F- TGAGGGTCTCAGTCTCGGTT R-TCCTCCAGAGAGACAGTTGAGA	*Zinc/iron permease* ZIP zinc/iron transport family
PNEG_01675 F-GTTCAGGAGATCAAGGGCGA R-CGAAAGCGCACTAGGAAACTG	*Zn(2)-C6 fungal-type domain-containing protein* GAL4-like Zn2Cys6 binuclear cluster DNA-binding domain; found in transcription regulators like GAL4. Domain consists of two helices organized around a Zn(2)Cys(Garcia-Santamarina and Thiele, 2015) motif; Binds to sequences containing 2 DNA half sites comprised of 3-5 C/G combinations.
PNEG_02322 F-GAAAGCACGACTTGCAGCG R-AAAGTGCATCAATCGGTCGG	*VWFA domain-containing protein* Von Willebrand factor type A (vWA) domain is made up of approximately 200 amino acid residues folded into a classic a/b para-rossmann type of fold. There are different interaction surfaces of this domain as seen by the various molecules it complexes with. Ligand binding in most cases is mediated by the presence of a metal ion dependent adhesion site termed as the MIDAS motif that is a characteristic feature of most A domains.
PNEG_01730 F-TGGGAAGGATGTGCAAAGGAG R-TCCACAATTCGGCCATTCACA PNEG_01236 F-ATGAGACGAAGAGGATCAGGTAG R-ACTTCGCTTACTTTCCGTGTG	*C2H2-type domain-containing protein* The C2H2 zinc finger is a classical zinc finger domain. C2H2-type zinc fingers often function as DNA or protein binding structural motifs, such as in eukaryotic transcription factors. C2H2 zinc finger proteins contain from 1 to more than 30 zinc finger repeats.
PNEG_01350 F-AGCAACGTTCAAGAAAAGGACG R-GCATTGCTCATGACGACCC	*Unspecified product* The catalytic domain of CAMK family Serine/Threonine Kinases. STKs catalyze the transfer of the gamma-phosphoryl group from ATP to serine/threonine residues on protein substrates. CaMKs are multifunctional calcium and calmodulin (CaM) stimulated STKs involved in cell cycle regulation. CaMKII is a signaling molecule that translates upstream calcium and reactive oxygen species (ROS) signals into downstream responses that play important roles in synaptic function and cardiovascular physiology.
PNEG_00014 F-GCAATGACACTGACTCCACG R-CCTGCGCATCCTTTCTGTTT	*FeS cluster biogenesis domain-containing protein* Fe-S cluster assembly iron-binding protein IscA
PNEG_01009 F-TATCCACCTCCTCCACCTCAAT R-TGCTGCCTTATTTGACGACGA	*Cysteine-rich transmembrane CYSTM domain-containing protein* Cysteine-rich TM module stress tolerance

### Extended treatment of *Pneumocystis murina* with copper

**Figure 4:**
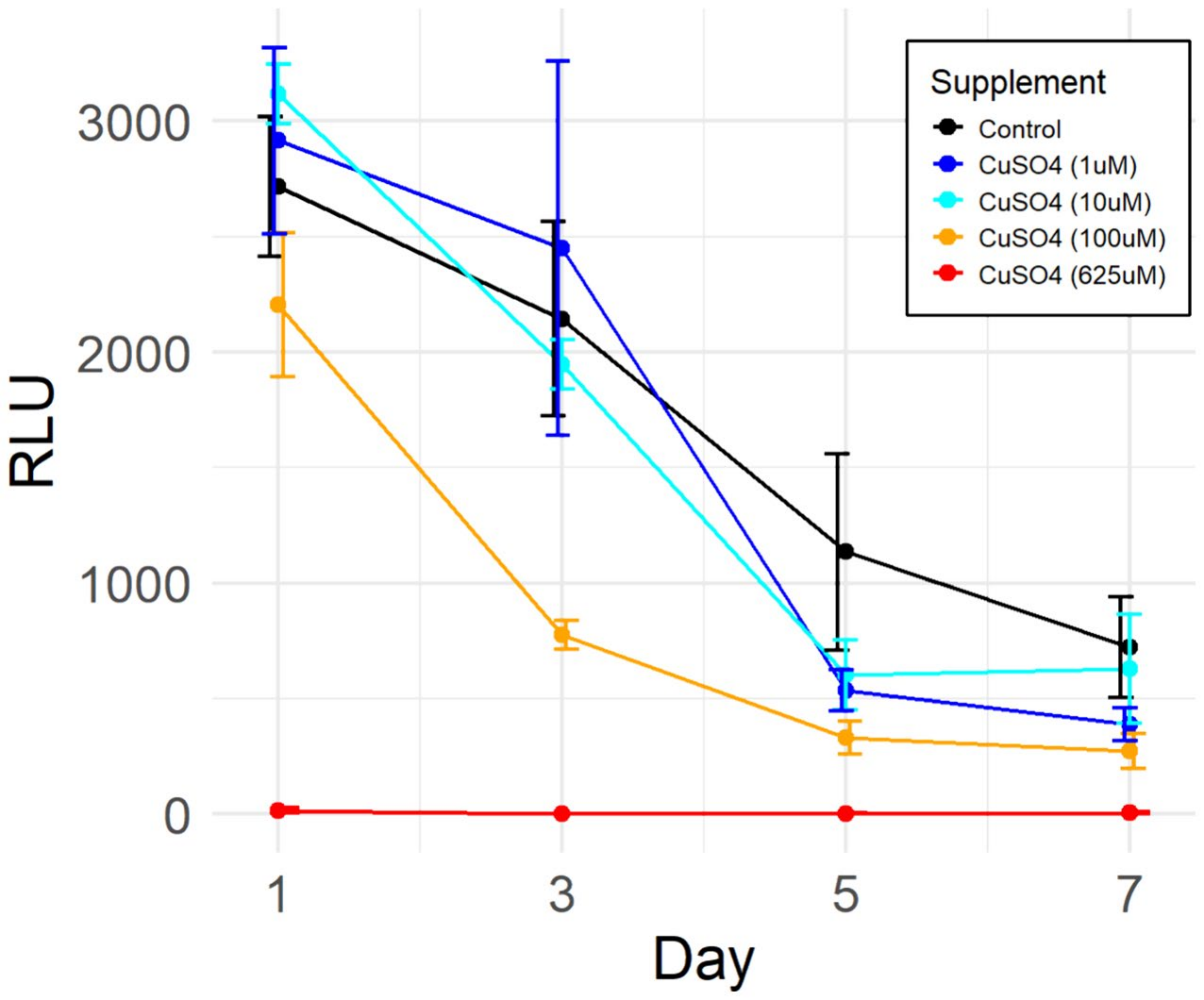
Efficacy of Cu(II) sulfate on *P. murina* viability. Viability assessed via ATP luciferase assay, indicated by relative light units (RLU) which correlate with the quantity of metabolically active organisms. Data derived from four biological replicates in each group. Adjustments made by deducting mean RLU of blank medium wells from all other values. Error bars represent standard deviations. Control comprises untreated organisms.

To assess the tolerance of *P. murina* to free labile copper, viability assays were conducted. Cryopreserved samples of the organisms were exposed to varying concentrations of Cu(II) sulfate for seven days, including both the concentrations employed in differential gene expression studies above and the concentration (625 μM) reported as beneficial in axenic culture media (Riebold et al., 2023). The results, depicted in Figure 4, reveal that *P. murina* exhibits comparable viability to untreated controls at lower CuSO_4_ concentrations (1 and 10 μM). However, at higher concentrations, Cu(II) sulfate demonstrates a toxic effect, indicated by a rapid decline in the number of metabolically active organisms.

The two-way ANOVA test reveals that both individual variables (days post-treatment and copper sulfate concentration) as well as their interaction exhibit statistically significant differences in results. Further analysis through Tukey’s post-hoc test demonstrates that treatments utilizing 100 μM and 625 μM CuSO_4_ significantly differ from the controls and treatments with lower concentrations. It should be noted that there is no continuous culture/growth method for any *Pneumocystis* species outside the host lungs. The decline in ATP in the short-term culture we used is typical for such *in vitro* systems.

## Discussion

This study explores the adaptive mechanisms of *Pneumocystis* species in a copper-rich environment. Due to their reduced genomic complexity, owing to an obligate parasitic lifestyle, these species exhibit a marked reduction (3–5 fold) in genes associated with copper binding (including sensing and tolerance) compared to other pathogenic and closely related free-living fungi (Table 1). Free labile copper exerts cytotoxic effects at elevated concentrations (Borkow and Gabbay, 2005; Valko et al., 2005). Higher organisms leverage this property for bactericidal and fungicidal purposes as part of innate immunity (Festa and Thiele, 2012; Djoko et al., 2015; Culbertson and Culotta, 2021), a phenomenon exemplified by the high concentrations of free copper in mammalian lungs (Garcia-Santamarina and Thiele, 2015; Besold et al., 2016). Our SEC-LC–MS/IMAC analysis of murine lung homogenates also showed that both infected and uninfected mice exhibited substantial levels of free labile copper in the lungs (Figure 1).

The identification of Cu-associated proteins and the genes differentially expressed under copper stress represents significant advancement in *Pneumocystis* biology. While prior studies have focused on Cu homeostasis in other clinically important fungal pathogens, such as *Candida albicans* (Schwartz et al., 2013; Khemiri et al., 2020), *Cryptococcus neoformans* (Ding et al., 2013; Festa et al., 2014), and *Aspergillus* species (Wiemann et al., 2017; Yang et al., 2018; Kang et al., 2020), our work offers unique insights into the much less-studied *Pneumocystis* species. Notably, we observed that significant gene expression changes in *P. murina* only occurred at elevated Cu(II) sulfate concentrations (100 μM, Table 5), suggesting a tolerance to lower copper ion levels. While it is established that mammalian lung environments can exhibit elevated copper levels, particularly under inflammatory conditions, we concede that our study did not directly measure these levels in infected lungs. Consequently, the copper concentrations used in our *in vitro* experiments (1, 10, and 100 μM) were chosen based on existing literature rather than empirical data from the specific conditions of Pneumocystis infection. This represents a limitation as these concentrations may not accurately mirror the *in vivo* environment.

Our study employed three orthogonal methods to identify Cu-related proteins in *P. murina*: sequence homology-based annotations, IMAC enrichment followed by proteomics, and RNA-seq analysis of gene expression changes after Cu exposure. Interestingly, these methods yielded largely non-overlapping results, with only four overlapping genes identified by the latter two approaches: PNEG_02587 (Hsp72-like protein), PNEG_03319 (Histidine kinase/HSP90-like ATPase domain-containing protein), PNEG_02584 (Hsp60-like protein), and PNEG_02989 (Major surface glycoprotein 2 C-terminal domain-containing protein). This discrepancy may arise from limitations in automated protein annotations based on sequence homology, which may not capture all protein molecular functions and binding sites, the low sensitivity and possible off-target selectivity of IMAC enrichment, or the constitutive overexpression of Cu-binding genes unaltered by additional CuSO_4_ treatment. Consequently, we recommend that future studies on *Pneumocystis* pathogenicity and survival under Cu stress consider all gene pools identified by these approaches.

The genes identified in our study provide the foundation for novel antifungal therapies targeting *Pneumocystis*-specific mechanisms of copper tolerance. This strategy is supported by literature indicating that disruption of copper uptake or detoxification processes compromises virulence in various fungal pathogens, such as *C. albicans* (Mackie et al., 2016), *A. fumigatus* (Cai et al., 2017), and *C. neoformans* (Waterman et al., 2007; Ding et al., 2013), underscoring the promise of targeting copper metabolism for antifungal interventions. Moreover, the conditional activation of copper ionophores, as demonstrated by the nontoxic boronic ester-masked 8-hydroxyquinoline derivative (QBP) against *C. neoformans*, illustrates a novel method for inducing copper-dependent cytotoxicity in fungal pathogens within host tissues (Festa et al., 2014). Collectively, these findings advocate for the exploration of copper homeostasis regulators as viable targets for antifungal drug development, aiming to discover and develop agents that specifically disrupt fungal copper regulation pathways while minimizing host impact, thereby advancing the treatment of fungal infections through a targeted approach to pathogen adaptation mechanisms.

Intriguingly, recent literature on axenic cultivation of *Pneumocystis carinii* recommends a medium composition (coined DMEM-O3) containing 100 μg/mL Cu(II) sulfate (Riebold et al., 2023). However, our CuSO_4_ supplementation viability assay indicates that such a concentration is exceedingly toxic, leading to rapid organismal death during *ex vivo* cultivation (Figures 4, 625 μM). This difference may stem from discrepancy in aliquot sizes used in the studies. We added a 0.25 mL aliquot of CuSO_4_ (625 μM) to the medium in the viability assay (see Methods), whereas the other study did not specify the actual amount of copper sulfate of the same concentration added to the culture medium. Another possibility in discrepancy is that *P. jirovecii*, the human-infecting species used in the other study, may have higher tolerance to the copper stress.

It should be noted that in contrast to other fungal pathogens affecting humans, *Pneumocystis* species are less studied and annotated due to challenges in cultivation and the application of traditional gene functional studies, such as knockouts or mutagenesis. Consequently, a significant proportion of their genes remain uncharacterized, with many proteins identified only as “uncharacterized,” “hypothetical,” or “predicted.” This limitation extends to Gene Ontology (GO) terms, as well as the cellular processes these genes participate in. Therefore, our ability to perform traditional functional enrichment analysis and identify enriched GO categories or biological pathways is hindered. Despite these obstacles, our study lays the groundwork for future research. As methodologies evolve and allow for the detailed study of these organisms, the genes we report could become focal points for understanding copper tolerance mechanisms and identifying new drug targets in *Pneumocystis*.

## Conclusion

This study provides new insights into the copper sensing and tolerance mechanisms of *Pneumocystis* species, with a particular focus on *P. murina*. Despite considerable gene loss compared to free-living fungi, *Pneumocystis* species have developed unique strategies for managing copper homeostasis. The discovery of specific copper-binding proteins and the differential gene expression in response to excessive copper exposure are significant contributions to the growing understanding of the adaptation mechanisms of extracellular pathogens in copper-rich environments, such as mammalian lungs. This preliminary study investigated *Pneumocystis murina* genes that may play a role in copper tolerance. These findings pave the way for future research into copper-binding proteins and their mechanisms of action once *in vitro* cultivation of the organisms becomes feasible. The findings also hold potential for the development of new therapeutic strategies targeting the distinctive aspects of *Pneumocystis* copper homeostasis.

## Data availability statement

The original contributions presented in the study are publicly available. This data can be found at: https://www.ncbi.nlm.nih.gov/bioproject/; PRJNA1076335.

## Ethics statement

The animal study was approved by Institutional Animal Care and Use Committee at the Veterans Affairs Medical Center, Cincinnati, OH. The study was conducted in accordance with the local legislation and institutional requirements.

## Author contributions

AP: Conceptualization, Data curation, Formal analysis, Funding acquisition, Investigation, Project administration, Software, Supervision, Visualization, Writing – original draft, Writing – review & editing. SS: Data curation, Formal analysis, Investigation, Methodology, Validation, Writing – original draft, Writing – review & editing. AA: Data curation, Investigation, Methodology, Resources, Validation, Writing – original draft. SR: Data curation, Formal analysis, Investigation, Methodology, Resources, Validation, Writing – original draft. JL: Data curation, Formal analysis, Investigation, Methodology, Resources, Validation, Visualization, Writing – original draft. MC: Conceptualization, Formal analysis, Funding acquisition, Investigation, Methodology, Project administration, Resources, Supervision, Writing – original draft, Writing – review & editing.

## Supplementary material

The Supplementary material for this article can be found online at:

https://www.frontiersin.org/articles/10.3389/fmicb.2024.1383737/full#supplementary-material

SUPPLEMENTARY FILE S1 Proteins identified by proteomics after the Cu-IMAC enrichment.

SUPPLEMENTARY FILE S2 Differentially expressed genes of *P. murina* exposed to CuSO4.

SUPPLEMENTARY FIGURE S1 PCA plots of the RNA-seq data from *P. murina* organisms exposed to CuSO4 over time durations of 0.5, 2, and 5 h. **(A)** The initial batch of treatment with CuSO4 at concentrations of 0 (controls), 1 ng/mL and 100 ng/mL. The organisms were derived from 6 animals. No differentially expressed genes were found. **(B)** The second batch of treatment with CuSO4 at concentrations of 0 (controls), 1, 10, and 100 μM over time durations of 0.5, 2, and 5 h. The organisms were derived from 5 animals, three of which (1, 5, and 6) were used in this study.
