# Supplementary figures and images for: Insights into copper sensing and tolerance in Pneumocystis species
Source: Front Microbiol. 2024 May 15;15:1383737. doi: 10.3389/fmicb.2024.1383737 (PMC11133566; doi:10.3389/fmicb.2024.1383737)

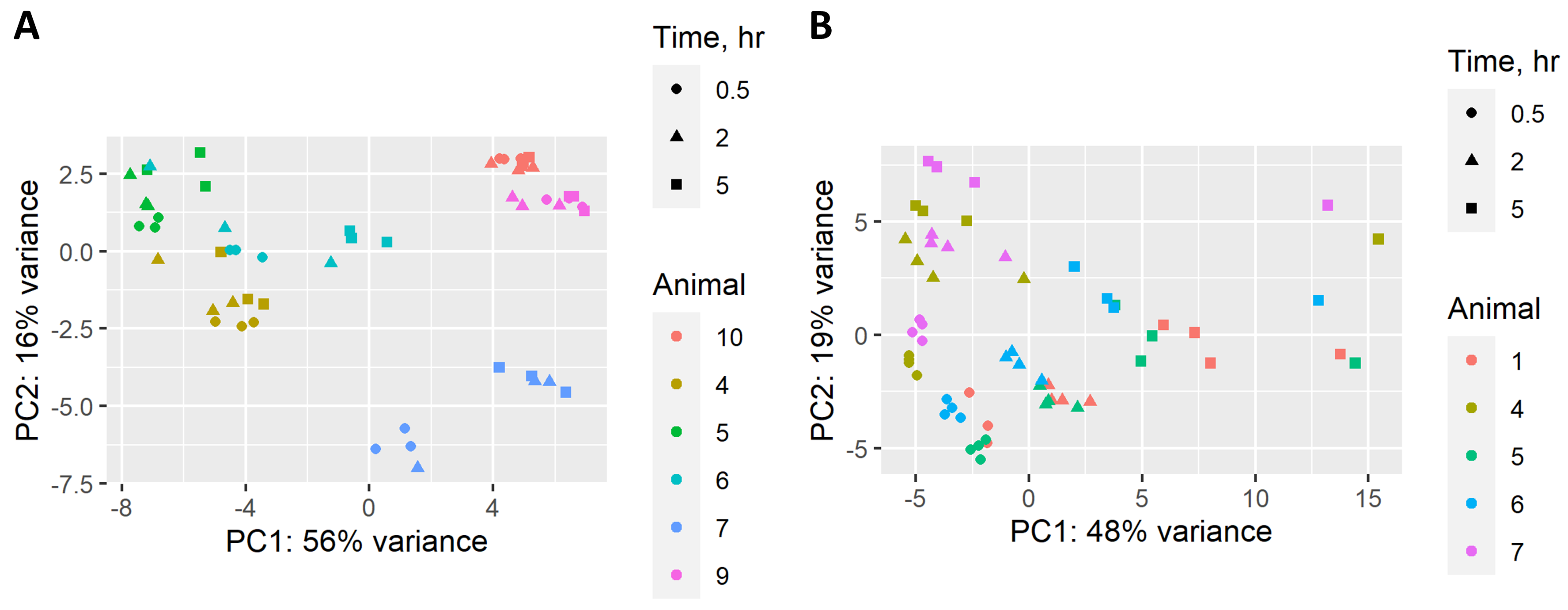

Supplement: SUPPLEMENTARY FIGURE S1 — PCA plots of the RNA-seq data from P. murina organisms exposed to CuSO4 over time durations of 0.5, 2, and 5 h. (A) The initial batch of treatment with CuSO4 at concentrations of 0 (controls), 1 ng/mL and 100 ng/mL. The organisms were derived from 6 animals. No differentially expressed genes were found. (B) The second batch of treatment with CuSO4 at concentrations of 0 (controls), 1, 10, and 100 μM over time durations of 0.5, 2, and 5 h. The organisms were derived from 5 animals, three of which (1, 5, and 6) were used in this study. [file Image_1.PNG]
